# The effects of titanium dioxide (TiO_2_) nanoparticles on physiological, biochemical, and antioxidant properties of *Vitex* plant (*Vitex agnus - Castus* L)

**DOI:** 10.1016/j.heliyon.2023.e22144

**Published:** 2023-11-10

**Authors:** Seyed Mostafa Moshirian Farahi, Mohammad Ehsan Taghavizadeh Yazdi, Elham Einafshar, Mahdi Akhondi, Mostafa Ebadi, Shahrouz Azimipour, Homa Mahmoodzadeh, Alireza Iranbakhsh

**Affiliations:** a Department of Biology, Science and Research Branch, Islamic Azad University, Tehran, Iran; b Applied Biomedical Research Center, Mashhad University of Medical Sciences, Mashhad, Iran; c Pharmaceutical Research Center, Pharmaceutical Technology Institute, Mashhad University of Medical Sciences, Mashhad, Iran; d Department of Biology, Payame Noor University, Tehran, Iran; e Department of Biology, Damghan Branch, Islamic Azad University, Damghan, Iran; f Faculty of Chemistry, Semnan Branch, Islamic Azad University, Semnan, Iran; g Department of Biology, Mashhad Branch, Islamic Azad University, Mashhad, Iran

**Keywords:** Titanium dioxide nanoparticles, Photosynthetic pigments, Phenylalanine ammonia lyase, Soluble sugars, Chlorophyll, *Vitex*

## Abstract

Titanium dioxide nanoparticles (TiO_2_NPs) are widely used in agriculture in order to increase the yield and growth characteristics of plants. This study investigated the effects of TiO_2_NPs on photosynthetic pigments and several biochemical activities and antioxidant enzymes of the *Vitex* plant. Different concentrations of nanoparticles (0, 200, 400, 600 and 800 ppm) at five levels were sprayed on *Vitex* plants on the 30th day of the experiment. TiO_2_NPs at different concentrations had positive effects on root and shoot dry weight and a negative effect on leaf dry weight. The amount of chlorophyll increased with the concentration of TiO_2_NPs; however, the amount of chlorophyll *b* showed a decreasing trend while the total chlorophyll had a constant trend. The highest amount of soluble sugar was obtained in the treatment of 200 ppm nanoparticles. The application of TiO_2_NPs did not have any effect on the content of proline and soluble proteins of *Vitex* plant. The effects of foliar TiO_2_NPs, compared to the control, showed a significant increase in the activity of antioxidant enzymes. In general, TiO_2_NPs had a favorable effect on dry matter production and some antioxidant and biochemical properties of the *Vitex* plant.

## 1 Introduction

With the rapid development of nanotechnology, engineered nanomaterials have also found an extensive usage in production of industrial, commercial, medical, diagnostic, and agricultural products [[1], [2], [3], [4], [5], [6], [7]]. Today, many industries use large quantities of nanoparticles, which enter the environment and the bodies of humans and animals in large quantities [[8], [9], [10], [11]]. These nanoparticles as well as their intermediate compounds interact with plants, animals, and other living organisms and impose positive and negative effects [[12], [13], [14]]. One positive effect of titanium oxide nanoparticles is enhanced crop growth. Studies have shown that the application of these nanoparticles can promote photosynthetic activity and nutrient uptake in tomato plants [15]. Additionally, titanium oxide nanoparticles can improve nutrient availability in soil. These nanoparticles increase the release of phosphorus from soil particles, leading to improved nutrient availability for plants [16,17]. Moreover, the high surface area of titanium nanoparticles allows them to absorb pollutants and filter wastewater, making them beneficial for environmental remediation purposes [18]. Biocompatibility of nanoagents is a crucial factor to consider when evaluating their safety. Studies have indicated that nanoparticles can have cytotoxic and genotoxic effects on different organisms. Thus, it is essential to thoroughly examine their behavior in terms of uptake, cytotoxicity mechanism, and both in vitro and in vivo toxicity [19]. Titanium oxide nanoparticles have been shown to induce cytotoxic and genotoxic effects on various organisms [20]. Furthermore, these nanoparticles can have adverse effects on aquatic organisms, causing oxidative stress and impairing their growth and development [21]. The effects of titanium oxide nanoparticles can vary depending on the specific conditions and concentrations. Further research is needed to fully understand the mechanisms underlying these interactions and to develop appropriate safety guidelines for the use of titanium oxide nanoparticles in various applications.

In recent years, application of titanium dioxide nanoparticles has been widely increased due to its biological properties and it is one of the most commonly used nanoparticles in agriculture [[22], [23], [24]]. Titanium is the ninth most abundant element in the earth's crust, and it can increase plant productivity by about 10–20% [25]. Nano TiO_2_ can improve light absorption, increase Rubisco and photosynthesis enzyme activity [26], increase nitrate absorption, accelerate the conversion of inorganic to organic material [27], and enhance the wet and dry weight of plants. Foliar application of TiO_2_ on Zea mays during the reproductive stage increased pigmentation and the yield [28]. Authors in showed that the use of titanium nanoparticles on spinach (*Spinacia oleracea*) caused the decomposition of organic compounds and higher hydrogen [29]. A study on the influence of foliar use of titanium nanoparticles on tomato (*Solanum lycopersicum* L.) showed that the nanoparticles increased the antioxidant enzymes properties [30].

Vitex is known as one of the rich sources of phytoestrogens [31]. The fruits contain essential oil, iridoid glycosides, flavones and flavonoids, diterpenoids, limonene and pinene [32]. *Vitex* is from the Lamiaceae family, which contains 250 species universal [33]. *Vitex* species are shrub types. It is one of the most important medicinal plants used in medicine and pharmacy in the world, which is found in the Mediterranean regions and central Asia as a wild flower along rivers and waterways [33]. Vitex's fruits are used to treat menstrual disorders, deficiency in corpus luteum, menopausal complications, and hyperprolactinemia [34,35].

Slow germination, slow growth, and related physiological parameters are the most important problems in the cultivation of this plant. Given the benefits of TiO_2_NPs, this nanoparticle has been used to improve the performance of this plant. The effects of these nanoparticles on the physiological properties of important medicinal herbs such as the *Vitex* plants need further studies. Nanoparticles seem to be able to affect some physiological processes and the antioxidant system of this plant. Therefore, the purpose of this work was to examine some biochemical and physiological traits of the *Vitex* plant treated with titanium dioxide nanoparticles under greenhouse conditions.

## 2 Materials and methods

### 2.1 Study materials and experiment

*Vitex* plant cultivation was accomplished in 2018 in the greenhouse of Islamic Azad University of Mashhad based on a completely randomized design with five replications. Each replication included of five pots and each pot contained 10 seeds of *Vitex* plant that was planted at a depth of 1 cm from the soil surface. The pots were placed on the platform under the sun and irrigation was applied on a daily basis. The greenhouse temperature ranged from 21 to 28 °C and the radiation was set as 16 h day and 8 h night and the relative humidity was maintained at 75 %. Plant seeds were attained from Dashtiar Isfahan Company. The concentrations of TiO_2_ NPs used in this experiment were 200, 400, 600 and 800 mg/L and distilled water as control treatment. TiO_2_ NPs were attained from Notirino Tehran Company. The samples were well ground and a homogeneous powder was obtained from the crystalline sample. In this way, it was possible to place a large number of crystal particles in the desired direction. The samples were mixed with a suitable adhesive and then molded. After that, the samples were analyzed by a XRD device (XRD Bruker D8 advance). In order to prepare the nanoparticle solution, 200, 400, 600, and 800 PPM of the nanoparticles were first placed on a shaker for 8 h, then they were mixed for 1 h by an ultrasonic device. Foliar application of nanoparticles as leaf spray by plastic sprinklers was performed on plants on the 30th day of the experiment. Non-treatment plants were also sprayed with deionized water. The experiment period was 65 days. After harvest, dry weight of stems, leaves, and roots were evaluated using a digital scale with an accuracy of 0.001 g.

### 2.2 Chlorophyll *a* and *b* assay

To measure the amount of leaf chlorophyll according to Ref. [36] method, after preparing the samples, the absorbance of the extract was documented using a spectrophotometer (Analytiic jena, SPEKOL 1500) at 645 and 663 nm and the amount of chlorophyll *a, b* and total chlorophyll was evaluated using the following formulas:

[12/7 (A663) −2.69 (A645)] *V/(w * 1000) = mg chlorophyll *a*/gram of fresh leaf.

[22/9 (A645) −4.68 (A663)] *V/(W * 1000) = mg chlorophyll *b*/gram of fresh leaf.

[20/2 (A645) +8.02 (A663)] *V/(W * 1000) = mg total chlorophyll/gram of fresh leaf tissue.

Where.

A = Light absorption of acetone extract, V = Final volume of acetone extract containing chlorophyll (ml) and.

W = Leaf fresh weight (g).

### 2.3 Measurement of soluble sugars in leaves

The content of soluble sugars from leaf, with some modifications, was measured using phenol sulfuric method [37]. One ml of the extract that was established in the previous section was mixed with 1 mL of 5 % phenol in a test tube and immediately 5 mL of concentrated sulfuric acid was added to it. Afterwards, the solution was stirred well and kept at room temperature for half an hour. Finally, the optical absorption of the final solution at 490 nm was measured using a spectrophotometer (UV/VIS Spectrophotometer, Jasco, 7800). A standard curve prepared with glucose was employed to estimate the concentration of soluble sugars.

### 2.4 Assessment of leaf soluble proteins and enzymes

To prepare leaf soluble protein extract, which was also used as an enzymatic extract, 250 mg of fresh leaf tissue was extracted with 1.5 mL of buffer (100 mM sodium phosphate buffer with 7.4 acidity) and grinded in cold porcelain mortar. After complete grinding and homogenization of the tissue, the resulting extract was centrifuged at 1500 g at 4 °C for 20 min. The resulting solvent was used to measure soluble protein content and enzyme activity [38]. Soluble proteins were measured through Bradford approach [39].

Peroxidase (POD) activity was measured according to the standard method [40]. After preparing the chemical solution, the extract was added and the changes in light absorption at 436 nm and the time when the light absorption increased from 0.05 to 0.1 (t) were recorded. Finally, the enzymatic properties were estimated using the following equation:

POD enzyme activity = (500) / (t)

The equation yields the activity of the enzyme per litter of extract from which the activity of POD enzyme in milligrams of fresh material can be calculated.

PAL enzyme activity was calculated based on the ability of the enzyme to convert phenylalanine to *trans*-cinnamic acid [41]. After preparing chemical solution, the light absorption of the specimens was documented at 290 nm with a spectrophotometer (Analytic gene, SPEKOL 1500). To determine the activity of PAL enzyme, the standard curve was developed by making different concentrations of trans cinnamic acid (10 concentration levels between 1 and 10 μg/mL) and the activity of the enzyme was reported based on the amount of cinnamic acid (μg) produced in fresh matter (gr).

To calculate the polyphenol oxidase enzyme activity, after preparing the enzyme extract, the test tubes containing the samples were placed in water (40 °C) and then 2.5 mL of phosphate 0.2 M, buffer solution, and 0.2 mL of pyrogallol 0.2 M solution were added to each test tube and temperature was increased up to 40 °C. At the time of reading the enzyme absorption, 200 mL of enzyme extract was added to each tube and immediately the changes in light absorption at 430 nm were documented by a spectrophotometer. Finally, to calculate the activity of polyphenol oxidase enzyme, the last absorption number was subtracted from the first record of absorption number and outcome was divided by four [42]. Proline was measured through Bates method [43] at 520 nm with a spectrophotometer (UV/VIS Spectrophotometer, Jasco, 7800). A standard proline curve was employed to define the proline concentration of the samples.

### 2.5 Extraction of malondialdehyde (MDA) and H_2_O_2_ from fresh plant tissue

In order to prepare an extract for measuring MDA and H_2_O_2_, 250 mg of plant fresh matter with 5 mL of 1 % trichloroacetic acid was finely crushed and ground. All extraction steps were performed on ice (4 °C). After crushing and complete homogenization of the tissue, the resulting extract was centrifuged at 10,000 g for 15 min (Sigma, Model 1–14) then the solvent was collected and used to measure MDA and H_2_O_2_ [44]. Optical absorption of samples for MDA was documented at 532 and 600 nm by a spectrophotometer (Analytic Jena, SPEKOL 1500). Light absorption at 600 nm is a non-specific absorption and must be subtracted from 532 nm. The value of MDA was measured using its extinction coefficient (155 mM^−1^cm^−1^) and reported in micromoles per gram of fresh weight. Light absorption was recorded for hydrogen peroxide at a wavelength of 390 nm using the Analytic Jena Spectrophotometer (SPEKOL 1500). The amount of H_2_O_2_ in the samples was calculated based on its extinction coefficient (280 mM^−1^cm^−1^) and reported in μmol/gr of fresh weight.

### 2.6 Statistical analysis

Data analysis was done by SPSS16 statistical software and the comparison of means was done based on ANOVA and Duncan's test. In addition, the graphs were drawn in EXCEL software.

## 3 Results and discussion

### 3.1 Characterization and physiological traits

**Fig. 1:**
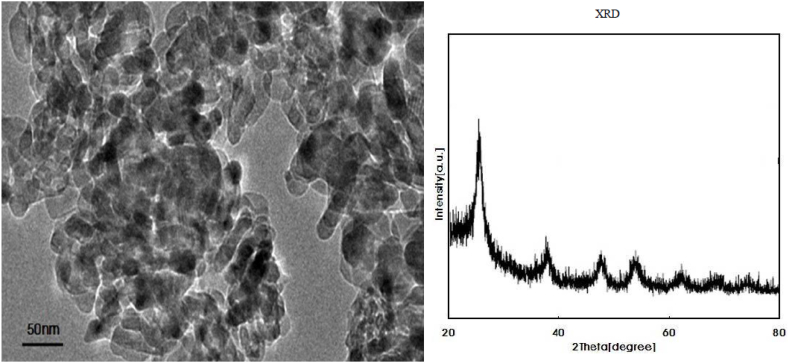
TEM image and XRD pattern of titanium dioxide nanoparticles.

TEM images of TiO_2_NPs (Fig. 1) revealed spherical shapes of NPs with particles sizes in the range of 20 nm. The XRD-diffraction peak clearly emerged as a sharp peak. The XRD pattern of the TiO_2_NPs (Fig. 1) at angles 25.5°, 35.5°, 44.5°, 52.5°, and 57.5° correspond to 101, 200, 004, 210, and 105 of TiO_2_NPs respectively.

Based on ANOVA results, employing titanium dioxide nanoparticles caused statistically significant changes in most of the physiological traits of the *Vitex* plant (Table 1). In Ref. [41], the effects of TiO_2_ NPs on spinach plants were examined under nitrogen deficiency conditions and normal conditions. The results showed that this nanoparticle was able to increase the nitrogen storage for the synthesis of chlorophyll and protein in spinach compared to the control and mass state of titanium dioxide, thus significantly increasing the growth of spinach. Hong et al. showed that chloroplast photosensitivity was reduced by TiO2 NPs [45], which is consistent with the results of this study. Titanium increases photosynthesis in two ways: one by changing the activity of proteins involved in photosynthesis, including fructose-1, 6 bisphosphatase, which affects Calvin cycle, gluconeogenesis enzymes, and changes in the pentose phosphate cycle, which play a role in carbohydrate metabolism. The second is through the increase of chlorophyll, which stimulates and increases photosynthesis [46,47]. TiO2 nanoparticles in rutile form can protect the structure of the chloroplast membrane against the reaction of oxygen free radicals and increase the activity of antioxidant enzyme systems such as POD and CAT [17,48]. Dehydrogenases are a group of enzymes with diverse effects on plants as reported by many researchers. These types of enzymes play a role in biological cycles and synthesis pathways of various compounds found in plants. For example, pyruvate dehydrogenase, malate dehydrogenase, isocitrate dehydrogenase, succinate dehydrogenase, and alpha-ketoglutarate dehydrogenase are dehydrogenases in the tricarboxylic acid (TCA) cycle and thus improving plant growth [49].

**Table 1:** Results of ANOVA tests for the effects of TiO_2_ NPs different concentrations on (*Vitex agnus*) studied parameters.

Root dry weight	Stem dry weight	Leaf dry weight	Chlorophyll *a*	Chlorophyll *b*	Total chlorophyll	Leaf soluble sugars
0.005	0.099	0.008	0.000	0.044	0.403	0.049
						
Proline	Leaf soluble protein	H_2_O_2_	MDA	Polyphenol oxidase enzyme	Phenylalanine ammonia lyase	Peroxidase enzyme
0.280	0.277	0.009	0.074	0.048	0.008	0.005

### 3.2 Dry weight of roots, stems and leaves

The consequences of variance analysis displayed that the effect of titanium dioxide nanoparticles on root, stem and leaf weight was significant (Table 1). The results of mean comparison (Table 2) show that in general, application of titanium dioxide nanoparticles had a positive influence on the dry weight of roots and stems and a side effect on the dry weight of leaves (Table 2). The results showed that root weight at different concentrations of application of TiO_2_ nanoparticles had the highest root dry weight, which was obtained in 600 and 800 ppm titanium oxide nanoparticles treatments. The lowest root weight was obtained from 200 ppm treatment, which was not significantly different from the control and 400 ppm treatments (Table 2). The maximum and minimum shoot dry weight were attained from 400 ppm and control treatments respectively (Table 2). Unlike stem and root weight, which increased due to the application of titanium oxide nanoparticles, leaf dry weight decreased at different concentrations, compared to the control (Table 2).

**Table 2:** Mean value of root, stem and leaf dry weight of (*Vitex agnus*) under different concentrations of TiO_2_ NPs.

TiO_2_ Concentration (ppm)	Root dry weight (mg (	Stem dry weight (mg)	Leaf dry weight (mg)
Control (0)	0.125 ± 0.02^b^	0.39 ± 0.48^b^	0.191 ± 0.05^a^
200	0.068 ± 0.01^b^	0.13 ± 0.02 ^ab^	0.117 ± 0.005^b^
400	0.033 ± 0.02^c^	0.45 ± 0.46^a^	0.105 ± 0.003^b^
600	0.093 ± 0.07^a^	0.39 ± 0.01 ^ab^	0.121 ± 0.011^b^
800	0.09 ± 0.04^a^	0.29 ± 0.34 ^ab^	0.119 ± 0.006^b^

Means followed by different letter(s) show significant difference at P < 0.05 significance level according to the Duncan's multiple ranges test.

Although studies have reported that wheat root length is not affected by TiO_2_ nanoparticles [50,51], our findings showed that the parenchyma and central cylinder density of roots were increased by exposure to TiO_2_ nanoparticles and this may explain the high root dry weight. Feizi et al. [50] also reported that the transfer of TiO_2_ nanoparticles to stems affected stem length, and its diameter with no effect on the root length. Phothi and Theerakarunwong [52] reported rice root length and biomass increase by using TiO_2_ nanoparticles. In other studies, TiO_2_ nanoparticles increased dry matter production of plants [[53], [54], [55]]. Another study showed that foliar application of TiO_2_ nanoparticles led to an enhancement in fresh and dry weight of plant through increasing the chlorophyll content, photosynthesis rate, and increasing Rubisco activity [29]. On the other hand, TiO_2_ nanoparticles can stimulate protein and chlorophyll generation by activating enzymes involved in nitrogen metabolism, thereby increasing the fresh and dry weight of plants [54]. Morteza et al. [28] studied foliar use of titanium nanoparticles (at concentrations of 0.02, 0.04 and 0.06 %) and the effects on biochemical properties and grain yield of cumin. They showed that the use of titanium dioxide NPs on Cumin, in comparison with the control group, had a positive influence on the biochemical properties of the plant and ultimately the use of this nanoparticle increased plant yield. Chen et al. attributed the biomass increase due to titanium dioxide nanoparticles stimulation to a higher mineral uptake in treated plants [56]. Titanium dioxide nanoparticles increase chlorophyll and photosynthesis, as well as the uptake of elements effective in chlorophyll production and photosynthesis such as iron, magnesium and nitrogen and thus result in higher plants growth.

In this study, leaf dry weight was reduced by application of nanoparticles, which was consistent with [57]. In a 16-day experiment on fenugreek treated with TiO_2_ nanoparticles, the researchers showed that the highest concentration of TiO_2_ nanoparticles reduced leaf area by up to 20 % compared to the control [58]. The reason for the decrease in leaf area and weight due to titanium oxide nanoparticles is its lack of direct interaction with the chlorophyll biosynthesis pathway and probably the decrease in chloroplast density [59]. Some studies have contributed the reduction of chlorophyll, carotenoids, and leaf aging to induction of an oxidative process in the plant [60], which may result in leaf weight loss under TiO_2_ nanoparticle treatments.

### 3.3 Chlorophyll *a*, *b* and total

**Fig. 2:**
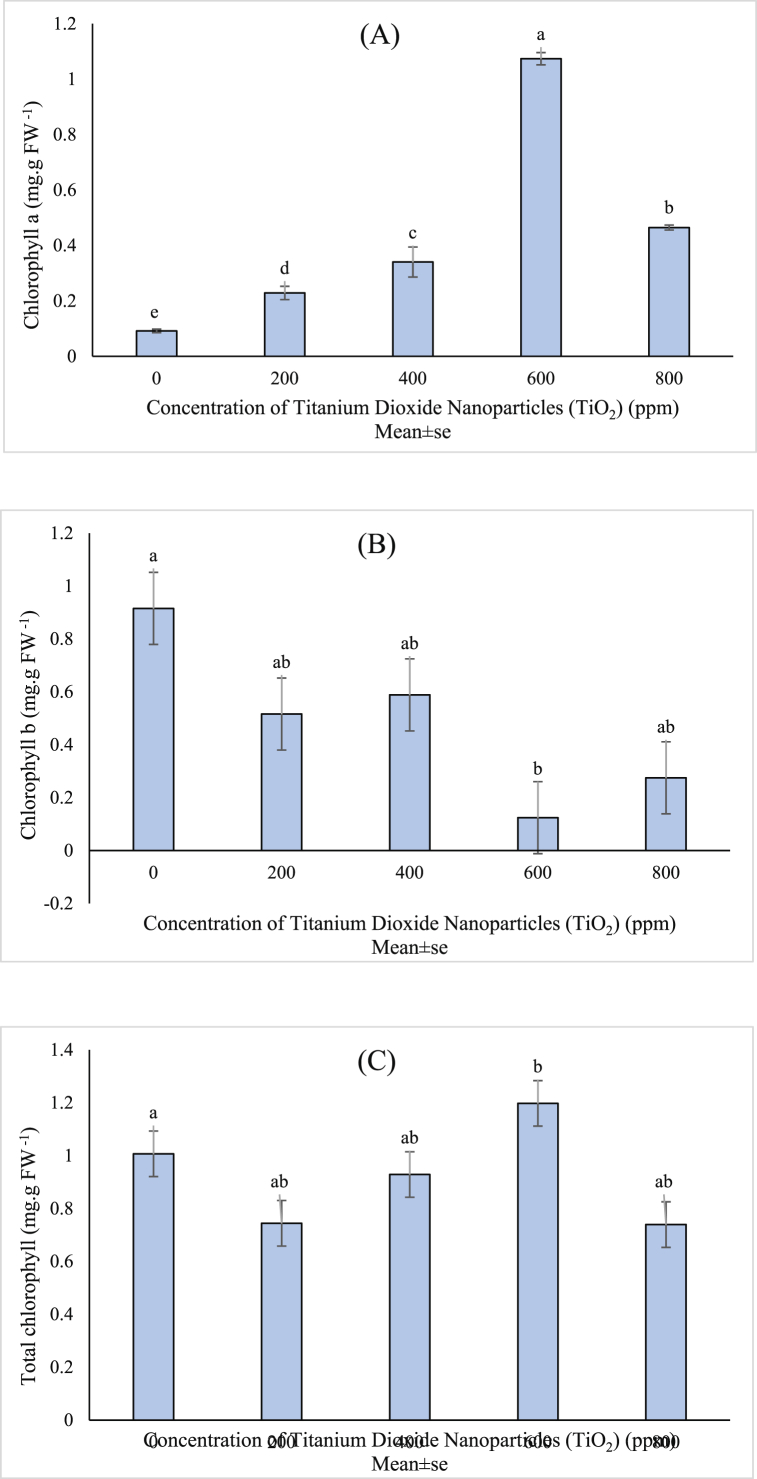
Mean value of TiO_2_ NPs concentrations effects on A) Chlorophyll *a*, B) Chlorophyll *b*, and C) Total chlorophyll content of (*Vitex agnus*).

The results of ANOVA showed that the effects of titanium dioxide nanoparticles on chlorophyll *a and b* content was noteworthy; however, the total chlorophyll content was not affected by these treatments (Table 1). Comparison of the mean values of chlorophyll *a* of different treatments showed that the nanoparticle increased chlorophyll *a* content and decreased chlorophyll *b* (Fig. 2a and b). However, since total chlorophyll is mainly composed of chlorophyll *a and b,* because of the different responses of the two types of chlorophyll the nanoparticles, the total chlorophyll content in different treatments did not show any significant difference (Fig. 2c). The highest amount of chlorophyll *a* (1.074 mg/g fresh weight) and *b* (0.915 mg/g fresh weight) were obtained in 600 ppm nanoparticle and control treatments respectively. The lowest amount of chlorophyll *a* (0.092 mg/g fresh weight) and *b* (0.124 mg/g fresh weight) were obtained in the control and 600 ppm treatment respectively (Fig. 2a and b).

Other studies have reported an increase in chlorophyll content because of foliar application of TiO_2_ nanoparticles [29,61]. In a study, TiO_2_ was used as a photo-protective substance, protecting the leaf surface from UV light and decreasing leaf sunburn damage [62]. These inconsistent results indicate that different plant species have different responses to different concentrations of nanoparticles. Cox et al. [63] stated that one of the reasons for the inconsistent results was different responses of TiO_2_ nanoparticles to UV light [63]. Qi et al. [64] described that nano-anatase intensely induce electron transfer, photosynthetic activity, O_2_ activation, and chlorophyll photophosphorylation activity under visible light and ultraviolet light. TiO_2_ nanoparticles increase light absorption by chloroplasts, which in turn activates the light-absorbing complex. This activation increases the photosynthetic capacity [65]. Titanium dioxide nanoparticles, by increasing photon absorption, stimulate the oxidation and redox which ultimately leads to increased photosynthetic capacity in the plant and prevents chloroplast aging [66]. Feizi et al. [50] showed that TiO_2_ nanoparticles caused chlorophyll synthesis, changes in dry weight, yield and some metabolic properties of photosynthetic organisms, which are often accompanied by induction of defense systems and a significant reduction in damage indices.

### 3.4 Soluble leaf sugars

**Fig. 3:**
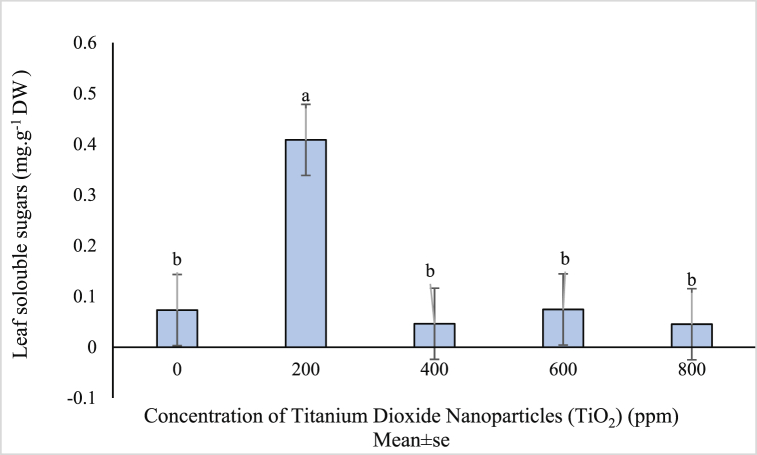
Mean value of TiO_2_ NPs concentrations effects on leaf soluble sugars of (*Vitex agnus*).

The results of ANOVA indicated a significant effect of titanium dioxide nanoparticles on the content of soluble sugars in the leaves (Table 1). Comparison of the mean content of soluble sugars in different treatments of TiO_2_ NPs indicated that only 200 ppm treatment had an enhanced soluble sugar content (0.408 mg/g leaf dry weight). There was no noteworthy difference between other treatments for the content of soluble sugars (Fig. 3). Because drought stress causes decomposes starch and remove it from the plant, raising soluble sugars is a regulatory response to drought stress [67]. Therefore, titanium dioxide nanoparticles can play a positive role in increasing the soluble sugars of *Vitex* plant and improving stress tolerance. In an experiment on the green bean plant, it was found that using 0.01 % of normal titanium dioxide nanoparticles increased soluble sugars [68]. The positive effects of iron nanoparticles on the increase of soluble sugars in *Citrullus lanatus* was also reported in Ref. [67].

### 3.5 Proline and soluble leaf proteins

**Fig. 4:**
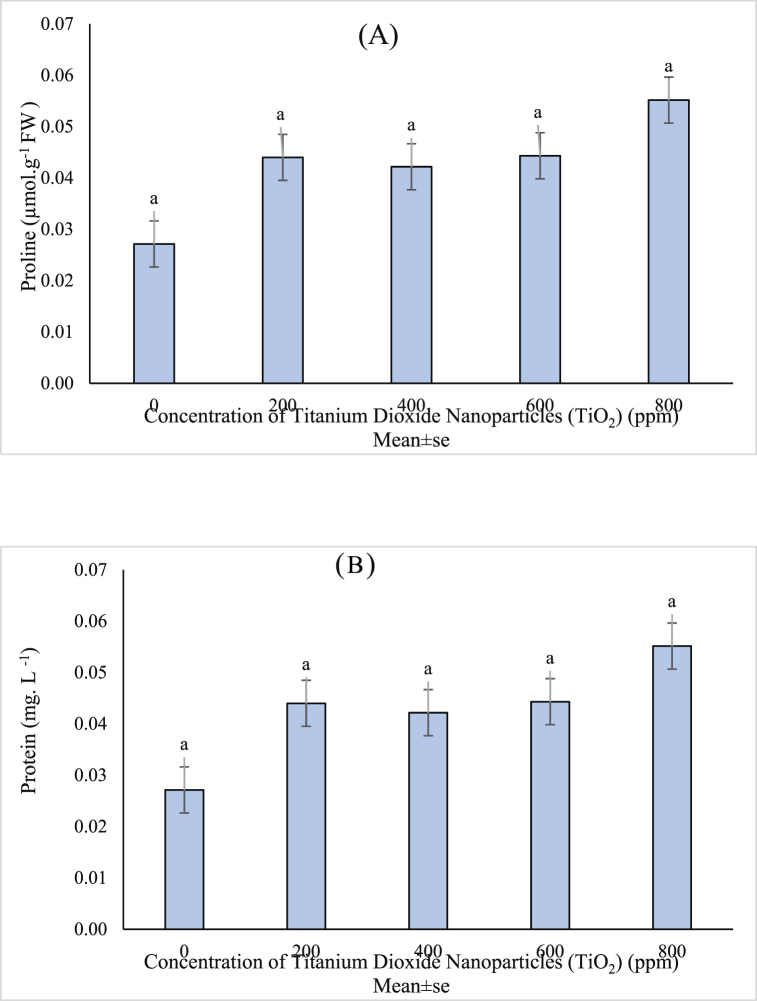
Mean value of TiO_2_ NPs concentrations effects on A) Proline and B) Protein content of (*Vitex agnus*).

The ANOVA results indicated that the effects of different concentrations of TiO_2_ NPs on the amount of proline and soluble leaf proteins was not significant (Table 1). However, comparing the means of proline content (Fig. 4a) and leaf soluble protein content (Fig. 4b) showed that the application of NPs caused a slight increase in proline and a slight decrease in soluble protein.

### 3.6 MDA and H_2_O_2_

The ANOVA results demonstrated that the effects of titanium dioxide nanoparticle concentrations on malondialdehyde content and hydrogen peroxide was significant (Table 1). Therefore, the content of MDA in different concentrations of nanoparticles was not significantly different from the control. MDA accumulation indicated lipid peroxidation, which often increases under stress. Koce et al. [69] exhibited that the use of TiO_2_ NPs did not significantly change lipid peroxidation and the antioxidant enzymes activity. These results are in contrast with the result of Song et al. [30] who used high concentrations of TiO_2_ nanoparticles (5000 mg/L) in their experiments.

**Fig. 5:**
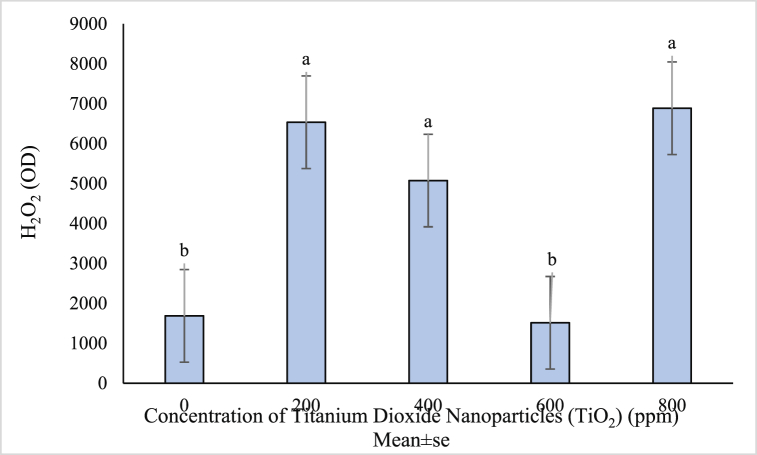
Mean value of TiO_2_ NPs concentrations effects on H_2_O_2_ content of (*Vitex agnus*).

The highest amount of H_2_O_2_ (6888.6) was obtained with 800 ppm nanoparticles treatment and the lowest amount of H_2_O_2_ (1514.7) was observed in the control (Fig. 5). Gao et al. (2018) reported that anatase titanium dioxide nanoparticles increased Reactive Oxygen Species (ROS) and cytotoxicity [70]. Consistent with the present study, Servin et al. [71] studied cucumber plant and found that hydrogen peroxide was increased in titanium nanoparticle treatment because of the oxidative stress caused by the nanoparticle. According to Aghdam et al. a notable enhancement in hydrogen peroxide was seen in some treatments, which was probably due to the strengthening of the cell electron exchange mechanism that reduced ROS production and MDA accumulation [72]. The use of NPs in appropriate amounts may either reduce H_2_O_2_ production or further activate H_2_O_2_ metabolizing enzymes [73].

### 3.7 Activity of polyphenol oxidase, phenylalanine ammonia lyase (PAL), and peroxidase (POD) enzymes

**Fig. 6:**
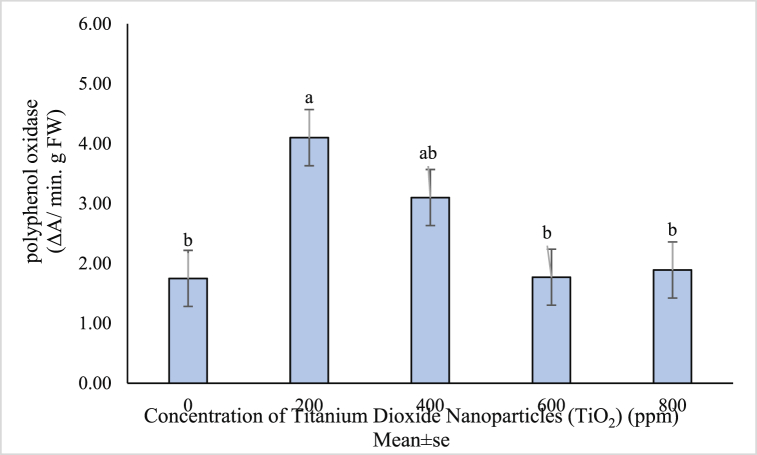
Mean value of TiO_2_ NPs concentrations effects on polyphenol oxidase enzyme activity of (*Vitex agnus*).

Increased activity of polyphenol oxidase enzyme is associated with plant defense mechanisms, including lignification and suberization through phenolic oxidation and their conversion into quinones and lignin formation in plant cells [74,75]. The ANOVA results showed that the effects of titanium dioxide nanoparticles on polyphenol oxidase activity was significant (Table 1). In general, the application of TiO_2_ NPs increased the activity of polyphenol oxidase enzyme. The highest enzyme activity was obtained at concentration of 200 ppm equal to 4.23 mg/g dry weight, and the lowest polyphenol oxidase activity was obtained at the control treatment equal to 1.38 mg/g dry weight (Fig. 6).

**Fig. 7:**
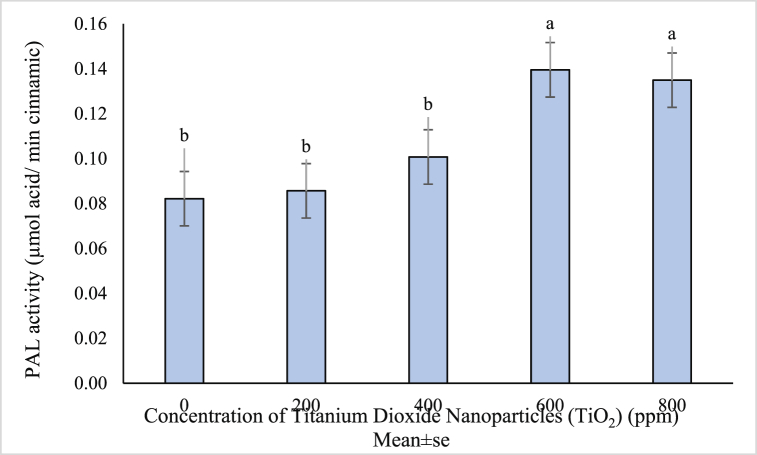
Mean value of TiO_2_ NPs concentrations effects on PAL enzyme activity of (*Vitex agnus*).

PAL is one of the main enzymes in the production of many phenolic secondary metabolites. The activity of this enzyme as part of the immune system is highly dependent on plant growth stage and environmental factors [76]. According to the ANVOA results, the effects of titanium dioxide nanoparticles on the enzyme activity was significant (Table 1). Application of TiO_2_ NPs also increased PAL activity. The highest enzyme activity at concentrations of 600 and 800 ppm was equal to 0.695 and 0.1349 respectively, and the lowest enzyme activity (PAL) was in the control treatment equal to 0.082 (Fig. 7).

**Fig. 8:**
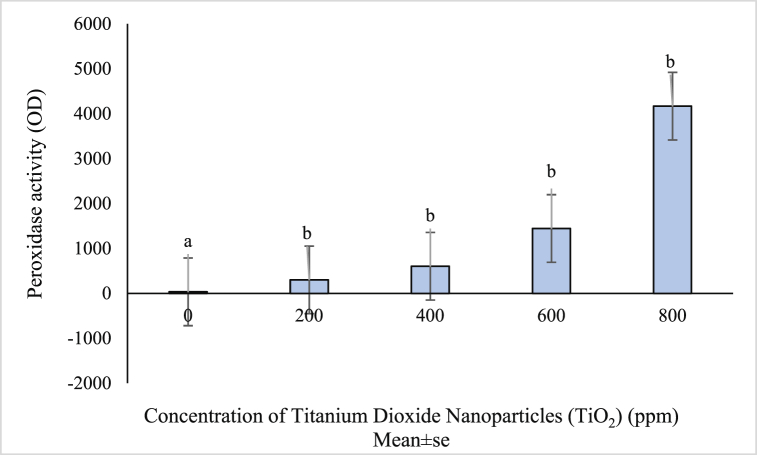
Mean value of TiO_2_ NPs concentrations effects on POD enzyme activity of (*Vitex agnus*).

Peroxidase is the main (backbone) enzyme in the antioxidant protection system. The ANOVA results showed that the effects of titanium dioxide nanoparticle concentrations on peroxidase activity was significant (Table 1). Comparison of the mean effect of various treatments of titanium dioxide nanoparticles on the activity of peroxidase enzyme showed that with an increase in nanoparticles concentration, the activity of this enzyme also increased. The highest enzyme activity was obtained with 800 ppm nanoparticle treatment and the lowest peroxidase activity was obtained with the control group (Fig. 8).

The destructive effects of ROS and lipid peroxidation yields are neutralized by the antioxidant protection system [77,78]. Titanium nanoparticles can increase antioxidant enzymes in plants. Increased activity of antioxidant enzymes by TiO_2_ nanoparticles has been reported in various studies [58,79]. Laware and Raskar [80] reported that the increase in peroxidase and catalase levels due to nanoparticle consumption of titanium dioxide depended on the nanoparticle concentration, while the activity of superoxide dismutase enzyme depended on the nanoparticle dose. Jacob et al. [81] described that treatments of 10–30 ppm of these nanoparticles enhanced the antioxidant enzymes activity in beans. Activation of the antioxidant system by TiO_2_ nanoparticles has also been described in *Spinacia oleracea* [82], *Zea mays* [28] and *Cicer arietinum* [83]. In addition, in a metabolic study on rice treated with this nanoparticle, an energy flow towards the synthesis of antioxidants was observed [84]. Nair and Chung [27] reported an enhanced peroxidase activity because of the application of copper nanoparticles (CuONPs) in soybean. Ali et al. (2019) reported an increase in antioxidant enzyme activity due to the use of silver nanoparticles on *Caralluma tuberculate* callus [85]. Increased PAL enzyme activity in *Annona muricata* L. due to foliar spray of silver and copper nanoparticles has also been reported [86].

## 4 Environmental concerns of using NPs in agriculture

Sustainable agriculture regularly relies on chemical fertilizers and components that have harmful effects on the environment [87,88]. Therefore, agrochemicals should be employed at lowest levels as possible to protect the environment. The nanoscience applications in agriculture can significantly improve the efficacy of agricultural inputs and hence they are a solution to preserve agroecosystems [[89], [90], [91]]. Different types of nanoforms such as nanoherbicides, nanofertilizers and the others have been established for agricultural usages. They could have considerable profits for agriculture such as enhancement of crops yields and removal of herbal pathogens. However, there are some concerns related to the nanoparticles applications in agriculture such as employment, uptake and internalization inside the plant cells. Hazardous effects of nanoparticles on herbs have been examined by several studies. It is known that silver nanoparticles could attach to the crop cell wall and cause alteration in color of treated crops and have a negative effects on growth levels [92,93]. Selenium (Se)-nanoparticles in high doses cause oxidative stress; while, Se-NPs are less toxic in comparison to bulk Se materials [94,95]. There are other reports that show that nanoparticles by binding to the roots of plants cause harmful effects in plants [[96], [97], [98]]. Therefore, in addition to the many and amazing applications of nanoparticles, the utilization of nanoparticles in agricultural usages should be with cautious, and more studies are needed in this regard to have the minimize the risks.

## 5 Conclusion

Foliar application of TiO_2_ nanoparticles in appropriate concentrations on *Vitex* plant can improve the production of dry matter through positive changes in soluble sugars, photosynthetic pigments and antioxidant enzyme activity. At 600 PPM, most of the physiological parameters were improved and therefore this concentration can be chosen as the best treatment. Few types of different nanoparticles have been tested on this plant so far; therefore, it is suggested to test different types of nanoparticles. This plant has a good resistance to drought stress, so it is recommended that drought stress is also applied. In some geographical areas such as Iran, this plant has little growth rate, and this nanoparticle can be used as foliar spray or as fertilizers with appropriate concentration and suitable conditions. This nanoparticle may play a role in regulating the phytochemical reactions of the plant as well. However, despite the favorable effect of this nanoparticle, it must be used with caution and great care to improve plant growth, because the effect of nanoparticles depends entirely on the dose, shape, and type of nanoparticles and plant species.

## Data availability statement

No data was used for the research described in the article.

## CRediT authorship contribution statement

**Seyed Mostafa Moshirian Farahi:** Writing – original draft, Software, Resources, Methodology, Investigation, Formal analysis, Conceptualization. **Mohammad Ehsan Taghavizadeh Yazdi:** Writing – review & editing, Writing – original draft, Resources, Methodology, Formal analysis. **Elham Einafshar:** Validation, Software, Methodology. **Mahdi Akhondi:** Resources, Formal analysis. **Mostafa Ebadi:** Methodology, Formal analysis, Data curation. **Shahrouz Azimipour:** Software, Formal analysis. **Homa Mahmoodzadeh:** Validation, Supervision, Methodology, Formal analysis, Conceptualization. **Alireza Iranbakhsh:** Writing – review & editing, Supervision, Project administration, Methodology, Data curation, Conceptualization.

## Declaration of competing interest

The authors declare that they have no known competing financial interests or personal relationships that could have appeared to influence the work reported in this paper.
