# Supplementary material for: The effects of titanium dioxide (TiO2) nanoparticles on physiological, biochemical, and antioxidant properties of Vitex plant (Vitex agnus - Castus L)
Source: Heliyon. 2023 Nov 10;9(11):e22144. doi: 10.1016/j.heliyon.2023.e22144 (PMC10685375; doi:10.1016/j.heliyon.2023.e22144)
Supplement: Multimedia component 1 [file mmc1.docx]

Glucose (mg/L)

OD

Y=0.008x+0.026 R^2^=0.992

Fig 1. Standard curve of soluble sugars.

OD

Gallic acid (mg/mL)

Fig 2. The standard curve of total phenol plotted based on specific concentrations of gallic acid.

Protein concentration (mg/mL)

OD

Fig 3. Standard curve of soluble proteins.

OD

Cinnamic acid (mg/L)

Fig 4. Standard curve of cinnamic acid.

OD

Proline concentration(mg/L)

Fig 5. Standard curve of proline.
